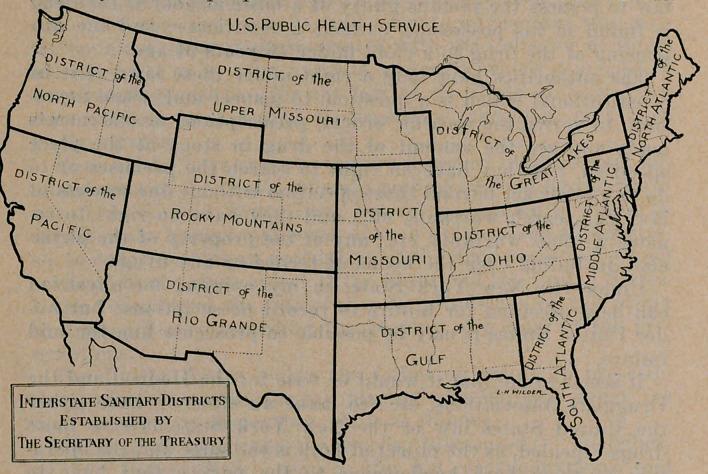# Topics of Public Interest

**Published:** 1915-08

**Authors:** 


### TOPICS OF PUBLIC INTEREST.

---

**(Relatively) Safe and Sane Fourth.** The Chicago Tribune has collected statistics showing that 9 persons were killed and 177 injured this year, as compared with 9 killed and 601 injured in 1914. Fire losses were $66,550 as compared with $76,035 in 1914.

---

**The Medical Library Association,** 1211 Cathedral St., Baltimore, aims to supply medical books and periodicals as needed by subscribing libraries and individuals. 1347 books and 2023 journals and pamphlets were distributed in the six months ending June 30. 945 books and 3142 journals were donated.

---

**War Relief.** The Rockefeller Foundation has issued a report of its work in Belgium, France, Serbia and Poland. $1,000,000 has been expended in Belgium beside $90,000 among refugees in Holland. $20,000 a year has been appropriated for stipends to scientific professors of Belgian universities for whom laboratory facilities have been provided in England. Except for equipping a hospital for Dr. Carrel, that research work might be done in addition to actual care of patients, it was found that no assistance was needed in France. Typhus and the threat of typhoid and cholera are acute in Serbia and about $125,000 has been expended, $85,000 contributed by the Foundation, the remainder by the Red Cross. $10,000 a month has been guaranteed by the Foundation for relief work in Poland. Practical results for humanity like these, reconcile us to having to pay a few cents a gallon for kerosene beyond the possible low price. And, as we have pointed out before, it is worth while to compare the rates charged for water with those for petroleum products. Recently, we have seen a spring water, possessing no special qualities, listed at 40 cents a half gallon. Of course, the suppression of numerous small businesses is quite another problem.

---

**Buffalo Tuberculosis Hospital.** The total of the low bids submitted for the proposed hospital on the West farm site, is $477,907, well within the limit fixed.

---

**Results of Narcotic Laws.** The possible immediate tragedies due to these laws are exemplified by the suicide of a man aged 28, after killing his mother aged 60, in Buffalo June 29. 11 similar deaths were recently reported for Chicago. The local health authorities have asked for an immediate appropriation of $2,000 to provide hospital care for victims of drug habits. It is not necessary that a single death should result from the inforcement of these laws although inevitable that many, finding it impossible to secure drugs as they have been in the habit of doing, will end their lives.

A curious side issue of these laws, is the indictment of a physician, not for direct violation of the law, but for harboring prostitutes and persons who are a nuisance by reason of drug habits, in his office. It has been considered one of the fundamental ethical principles of the medical profession that relief was to be afforded in case of need, without regard to political, religious, personal or social objection. However much we may desire nice, respectable persons for a clientele, it would be a rejection of humanitarian principles to imply in any way that the most unfortunate and degraded human being may not freely seek professional advice and aid.

Another important phase of the narcotic laws, both in their immediate and their ultimate effects, is legislation by ruling. For example, it has been ruled that "personal administration" does not cover the attendance on a patient at the physician's office, although it does at the patient's residence. We are not informed just where "personal" ceases to mean personal, whether, for example, attendance on a person taken sick at a hotel or on a street car, or injured on the street is personal or not. More recently, it has been ruled that a proprietary mixture is a preparation and a prescription not. Such rulings remind one of the old story of two travelers, one with a cat and the other with a bat, on a railway train in England, the regulations of the company specifying only the payment of a special fare for dogs. After consultation, the conductor issued the edict that cats were dogs and paid a fare, that bats were insects and went free. There used to be a fiction among political economists that the functions of a democratic government were strictly divided among executive, legislative and judicial authorities. The most learned judges have always shown a reluctance to tamper with laws as formulated but we now have bureau clerks making their own laws, contrary to common sense and the dictionary.

---

**High School Graduates.** The four high schools of Buffalo graduated 655 pupils this year. Persons of the average of high school graduates comprise 1.96% of the total population, on the average—about 8950 for Buffalo. Thus about one in thirteen of the rising generation receives a full high school education, approximately three times as many as in 1880.

---

**A National Medical Licensing Board.** The theoretic desirability of national medical licensure has been considered impracticable on account of the peculiar conflict of state and national authority in our government. In recent years it has been shown that ''the Constitution stands for little among friends'' in a salutary sense as well as that of the politician. Dr. Rodman in his address before the A. M. A. formally advocates a national board comprised of the Surgeon Generals of the Army, Navy and Public Health Service, with representatives from the Confederation of State Boards of Examiners, the Association of American Medical Colleges, the American College of Surgeons and the A. M. A. Even in the lack of constitutional authority, the states could and probably would, under sufficient influence, pass laws accepting the certificate of such a board.

---

**The Mayo Foundation.** After considerable discussion and some modification of the original plan, the regents of the University of Minnesota—which is a real institution of learning and not such an organization of education as in N. Y.—have accepted the generous gift of the Drs. Mayo. The scientific work will be carried on in a building to be erected in Rochester, in which the University will have the right to space for teaching purposes. Clinical privileges will also be granted to students. After Sept. 1, 1921, the principal of the endowment, amounting to $1,500,000 and some accumulated interest, beyond current expenses, will become the property of the University absolutely.

---

**Addition to Perrysburg Tuberculosis Hospital.** Contracts have been let for the erection of pavilions for the J. N. Adam Memorial Hospital, to increase the accommodations from 160 to about 300. By crowding, 203 patients are now cared for. It is expected that the additional pavilions will be ready before Sept. 1.

---

**The Tubercular Death Rate** has decreased from 326 : 100,000 population in 1880 to 146.6 in 1913, according to George M. Cooper of Washington.

---

**The Speeding Ambulance.** A Buffalo ambulance, on a call to a moderately severe accident, has recently killed three persons. A few days later another collision occurred without fatalities. It is now proposed to withdraw the exemption from traffic regulations. In our experience, the difference between the maximum speed at which a vehicle can travel, providing that nothing gets in its way, or that a moderate attempt is made not to strike an obstacle dangerous to the vehicle itself, and the speed, varying with circumstances, but always insuring reasonable control, is about one minute per mile. Allowing five miles per round trip, for the average city ambulance call, and granting that the surgeon and instruments are provided for operation immediately on the arrival of the patient, it will still take some time before the five minutes extra time per case, will have saved three lives. A few months ago, we published a similar report, from Boston, with some comments which were intended to have a practical application.

---

**Municipal Reporting of Drug Habitues.** The Health Dept. of Buffalo has requested an ordinance requiring physicians to report users of drugs.

---

**Narcotic Laws.** Pursuant to request, I hereby, to the best of my ability, give for the benefit of physicians, a statement of what in my opinion is the law of New York State and United States re coca leaves and its derivatives, opium and chloral and their derivatives:

Coca Leaves, Etc., New York State Law.—(b) (c). Physicians shall upon delivery of cocaine or its salts, etc., make a record in a book kept for that purpose, of all purchases of the same, stating the date of purchase, the quantity purchased, the name and form in which it is purchased, the name and address of the seller, the name of the person by whom the purchase is made, the name of the person by whom the entry is made, a description of the package or container in which the substance was purchased, and a statement that such purchase is sold and purchased in the original package; that the package was sealed, that the seals thereon were undamaged and unbroken, and that the labels were attached thereto, said labels distinctly displayed the name, and quantity of cocaine or its salts, alpha or beta eucaine, or their salts, and the word "poison" with the name and place of business of the seller, all printed in red ink, and that said labels were not in any manner defaced or damaged, a statement showing how delivery was made, whether personally (or by mail, which Postal laws forbid), express, freight, or messenger, and also an entry shall be made in said book of the particular place in which such substance purchased is to be kept by the purchaser, which place shall not be changed without making a change in the entry of said book opposite the original entry, to be signed by the purchaser. This record and statement must be signed by the purchaser and kept in his regular place of business and shall be open for inspection at all times by proper officers, and be preserved for five years.

A physician may carry for use in his profession such drugs or compounds thereof, providing the amount so personally carried and the amount kept in the place scheduled in his record shall not together exceed a total of one and one-eighth ounces of such drugs.

A physician after **personal** examination of the patient may prescribe and himself dispense such substance to a patient provided he execute and deliver the certificate required of the dispensing druggist, to wit, a certificate giving name, age and address of person furnishing, of the physician prescribing, date of furnishing and amount of drug.

No physician shall possess more than one and one-eighth ounce of the substance.

If a physician issuing a prescription desires that the use of the substance to be for a longer period than ten days, I should gather he must state that fact in said prescription, as it must be so stated in the certificate.

A physician may prescribe after personal examination any of the said drugs in any amount. There appears to be no limit, but the United States has made a ruling which is set forth hereafter.

A physician shall keep a record in a book kept for that purpose of all alkaloid, cocaine, or their salts, eucaine or its salts or a mixture thereof, disposed of by him, and the difference between the amount so recorded plus the amount in the place designated, and the amount received, shall be evidence of violation of this section, and he shall balance the book with amount on hand at least once in six months.

Chloral and Opium and Its Salts, Alkaloid, Etc. New York State Law Amended April 17, 1915.—A physician may prescribe any of these drugs for use of a patient in any amount, no limit. But see United States ruling.

The prescription must contain substantially the following: The name in full of the physician, his office address, the name, age and address of the person to whom and the date on which such prescription was issued.

Said prescription can only be issued after **physical** examination of the party for the treatment of disease, injury, or deformity. A physician who dispenses these drugs shall at the time of dispensing of the same, place upon the package a label or deliver therewith a **certificate** stating the name and address of the person selling or furnishing same, to wit, himself, the name and address of the physician, himself, date of sale, and the name of the person to whom such sale was made. All orders for the purchase of any of the drugs must be made on special order blanks which used to be furnished by the Health Department, which Health Department now authorizes the use of the United States blanks to be bought in books of ten for ten cents from Collector of Internal Revenue. All physicians shall keep on record the name and address of each person to whom these drugs are dispensed, given away or in any manner delivered and the quantity so dispensed, given or delivered, whether such disposition be in the nature of compounds or otherwise, and if in the preparation of compounds, the quantity so used in each compound.

Such record to be preserved for two years. No physician shall dispense, sell or give away any of these drugs to any child under the age of sixteen years. Violation a felony.

United States Law in Effect Jan. 15, 1915, re Opium and Coca Leaves, Their Salts or Preparations.—Every physician must register with the Collector of Internal Revenue of the district his name, place of business, and where his business is to be carried on and pay said collector annually a tax of $1.00 per annum.

Physicians may prescribe, dispense and distribute said drugs to a patient, but must keep a record of all his drugs dispensed, distributed or **prescribed** according to the ruling hereinafter mentioned, showing the kind and the amount dispensed, distributed or prescribed, the date and the name and address of the patient. The only exception is that the physician does not have to keep a record of what is dispensed, distributed or prescribed when he personally visits the patient. This record shall be kept for two years.

The physician shall when giving an order to the seller for any of said drugs, make a duplicate thereof on a form issued in blank and shall preserve such duplicate for two years. All order forms, prescriptions and statements are open to the inspection of the proper authorities charged with the enforcement of any law.

The provisions of this act do not apply to dispensing remedies and preparations containing not more than two grains of opium, or one-fourth grain of morphine, or one-eighth grain of heroin or one grain of codeine, or any sale of them in one fluid ounce or in liniments or ointments for external use.

Resume.—Every physician must obtain these drugs only on a written order made in duplicate on blanks to be bought from the United States Collector of Internal Revenue, which blanks explain themselves.

Every physician should at the time of delivery keep a record in separate books of the purchase of coca and its derivatives and compounds, and of opium and its compounds, and enter in said book the place where the drug is to be kept. New York State Law requires a full statement of the coca delivered, the particulars of which are set forth above.

Every physician shsall keep, in a suitable book (United States ruling) for recording, the date when, the kind and the quantity of, and the name and residence of the patient to whom, these drugs are dispensed, distributed or prescribed (except when be visits).

Every physician shall keep a carbon copy or duplicate of every prescription (United States ruling), which he gives of all these drugs, and a written record in a book of what he dispenses of these drugs, according to the form set forth in the paragraph above.

Every physician shall give to whom ever he dispenses a certificate setting forth the name and address of the physician prescribing, the name and address of the person furnishing, and the name, age and address of the patient and the date and the amount furnished, and it should be signed by said physician and he should keep a duplicate thereof. This is required by the United States Law and the rulings.

Section 6 of the United States Law seems to cover solutions containing small quantities of opium and its salts, and says that the provisions of this Act do not apply to them, but it does not cover coca and its salts, and I would advise that this matter be taken up by those interested with the Collector of Internal Revenues as to what entry is necessary where it is used in very small quantities; for the law of the State of New York does not call for a record of disposition, except of coca once in six months.

None of the laws seem to require a record of the actual administering of the drugs, unless it may be covered by the wording of the United States Law, but in my opinion it would be safe to make a short statement of such drugs, when administered, in order to show an accounting of the drug possessed, which seems to be the main object of the Statute of the United States. I also recommend the observance by physicians of the ruling by United States regarding the keeping of a copy of the prescriptions.

The United States makes the following ruling re prescriptions, which accounts for my placing prescriptions under the same heading as dispensing. It is as follows:

"This office construes the words "dispensed," "distributed," or "prescribed," used in the Act, as synonymous, and that a physician, dentist or veterinarian "dispenses" within the meaning of the law when he writes a prescription calling for any of the narcotic drugs to be filled by a registered dealer." In effect May 11, 1915. This ruling may be ultra vires.

No law limits the amount to be prescribed or dispensed by physicians, yet the United States has ruled as follows, though admitting that the law does not limit the amount to be dispensed or prescribed at one time:

"Where a physician, dentist, or veterinarian prescribing any of the aforesaid drugs in a quantity more than is apparently necessary to meet the immediate needs of a patient in the ordinary case, or where it is for the treatment of an addict or habitue to effect a cure, or for a patient suffering from an incurable or chronic disease, such physician, dentist, or veterinarian surgeon should **indicate on the prescription** the purpose for which the unusual quantity of the drug so prescribed is to be used. In cases of treatment of addicts these prescriptions should show the good faith of the physician in the legitimate practice of his profession by a decreasing dosage or reduction of the quantity prescribed from time to time, while, on the other hand, in cases of chronic or incurable diseases such prescriptions might show an ascending dosage or increased quantity."

The violation of these are misdemeanors.

The only felonies are: when a physician fails to give a certificate to the patient, because every person not authorized by law to possess the same is guilty of a misdemeanor if the drug is found in his possession without a certificate; and the dispensing of the drug to a child under 16 years of age.

The authorities have only a right under these laws, if it be constitutional which is a question, to inspect and, when necessary, to verify the records, orders, prescriptions or statements made and see the amount of the drug in stock at the place specified, but they have no right to search the premises or to do any other act beyond those specified without due process of law, i.e., search warrants, etc., and they have no right to remove without warrants, etc., any of the property of the physician, including even the books of record or any drugs.

Under the New York State, in my opinion, no physician can be prosecuted for failure to record prescriptions; but under United States it may be possible to prosecute him for said failure.

It seems to me that it would be wise for the Medical and the Druggists Associations, etc., to make an effort to have either the United States law or the New York State law re these drugs repealed, as the object of both is the same and the operation of them both burdensome to the parties that have to handle the said drugs.

The laws on this subject are so complicated that the above is the best interpretation thereof that I can now give owing to the shortness of time for my consideration thereof.

ALFRED L. HARRISON,
Attorney for Erie County Medical Society.

Dated, June 22, 1915.

---

**Dental Dispensary for Rochester.** Mr. George Eastman has donated $300,000 for establishing a free dental dispensary and will contribute $30,000 a year for five years for maintenance. Private citizens are to contribute $10,000 a year for the same period. If successful, Mr. Eastman will then endow it with $750,000 on a permanent basis.

**Thefts of Narcotics.** One of the developments of the narcotic laws is the theft of drugs by habitues. Dr. Duchscherer of Buffalo, reported the theft of 50 1/4 grain morphine tablets by a man pretending to be a patient awaiting his return.

**Increase in Automobile Licenses.** The Buffalo office has issued 64,557 licenses up to the middle of July—an increase of 12,410 over the same period of 1914. About 12,700 licenses are for automobiles owned in Buffalo.

**Interstate Sanitary Districts.** The accompanying map, furnished by the Surgeon-General, shows the division of the country for the purposes of the U. S. Public Health Service.

## Figures and Tables

**Figure f1:**